# Health-related quality of life measures as predictors for recurrent hospitalization and mortality among patients in heroin-assisted treatment

**DOI:** 10.1007/s11136-025-04019-5

**Published:** 2025-07-16

**Authors:** Francesca Melis, Timo L. Kvamme, Christian Tjagvad, Desiree Eide, Birgitte Thylstrup, Thomas Clausen, Morten Hesse

**Affiliations:** 1 Institute of Clinical Medicine, Norwegian Centre for Addiction Research (SERAF), University of Oslo, Oslo, Norway; 2 Centre for Alcohol and Drug Research, Tuborgvej 164, Copenhagen, 2400 NV Denmark

**Keywords:** Opioid use disorder, SF-36, Opioid maintenance treatment, Latent class analysis, Hospital utilization, Trajectories, Heroin-assisted treatment

## Abstract

**Purpose:**

Individuals with opioid use disorder face complex health needs often necessitating hospital-based care, and many reported low health-related quality of life (HRQoL). This study assessed HRQoL factors as predictors of recurrent hospital-based care and mortality among heroin-assisted treatment (HAT) patients, identified patient groups with distinct HRQoL trajectories, and compared their hospital contact hours and mortality rates with the general population.

**Methods:**

A longitudinal cohort study was conducted, involving 541 HAT patients in Denmark from 2010 to 2018, along with a matched sample from the general Danish population. Using recurrent event regression and latent class growth analysis, we analyzed predictors of recurrent hospitalization and established trajectory groups. HRQoL was assessed using the 36-Item Short Form Health Survey (SF-36) and linked to multiple national registry data which provided demographic variables, hospital utilization, and mortality data.

**Results:**

“General health” emerged as the largest and only significant predictor of recurrent hospitalization. Mortality was predicted by age and ‘role limitations due to emotional problems’. We identified four trajectory groups based on “general health ”, showing either a decreasing or stable trajectory. Groups differed significantly in terms of average hospital contact hours and mortality and all groups had lower ‘general health’ scores and greater mortality than the representative Danish sample. The most severe group had four times higher usage of hospitalization hours.

**Conclusion:**

SF-36 ‘general health’ is a key risk stratification tool for HAT patients predicting outcomes like hospitalization and mortality. Identifying high-risk individuals can help clinicians implement timely (somatic) care, particularly for long-term patients.

**Supplementary Information:**

The online version contains supplementary material available at 10.1007/s11136-025-04019-5.

## Abstract (unstructured)

People with opioid use disorder, in particular those in heroin-assisted treatment (HAT), often experience serious health challenges, frequent hospital visits, and a lower quality of life compared to the general population. Understanding their health-related quality of life (HRQoL) is essential for improving care and outcomes for this vulnerable group. The key issue this study addresses is whether patients’ own report of HRQoL can identify those who will need more healthcare and die prematurely. We assessed aspects of HRQoL associated with recurrent healthcare contacts and mortality, and constructed groups based on the development of their HRQoL over time. “General health” was the strongest and only predictor of repeated hospital contacts, while age and “role limitations due to emotional problems” were the only predictors of mortality. We found four distinct HRQoL trajectory groups among HAT patients. Three groups deteriorated in HRQoL, and one was stable. All groups utilized more health services and were at higher risk of dying compared to the general population. The group with the most severe HRQoL challenges used hospital resources four times more frequently. In summary, measuring the “general health” factor is essential for predicting hospitalizations. These findings highlight the importance of implementing targeted interventions to enhance healthcare for the most at-risk individuals in this population, particularly addressing somatic health issues.

**Supplementary Information:**

The online version contains supplementary material available at 10.1007/s11136-025-04019-5.

## Introduction

Individuals with opioid use disorder (OUD) typically experience several acute and chronic health conditions that often require medical attention in a hospital setting [1–6]. Due to the particular intersection of multiple substance use disorders [4, 7, 8], physical and psychiatric comorbidities [7, 9–11], individuals with OUD record high rates of hospitalization and recurrent admissions [8, 11–17]. In particular, people who inject drugs demonstrate high healthcare use. In one study, 78% had at least one primary care visit within a year (8 visits median) and 60% an ER visit within 2 years [12]. Among inpatient OUD patients, nearly 1 in 5 were readmitted within 30 days, and about 1 in 3 within 90 days [16]. More in general, substance use disorder diagnoses are associated with more frequent acute care events within 30 days (average 0.63 vs. 0.32 per patient without diagnosis), and a higher readmission risk (33% vs. 23%) [8].

This reocurring pattern of hospital use has been referred to as a “revolving door” phenomenon [18]. It reflects a subgroup of high utilizers, i.e., patients responsible for a disproportionate share of healthcare services and costs. Common factors in the OUD population, such as high rates of mental health diagnoses, negative social determinants of health, as well as the severity of OUD-related dysfunction, are associated with (re)hospitalization and contribute to the ‘high utilizer’ patient status [17, 19–22]. Among individuals with subtance use disorders, the likelihood of early readmission increases with multiple chronic conditions (17.0% no chronic conditions to 82.8% in those with 4 or more) [11]. Unemployment and unstable housing also shorten the readmission time [22]. High utilizers of healthcare have a 2–3 higher mortality rate [19] and post-hospital discharge is a critical period for increased mortality, particularly for older individuals with OUD [23].

Opioid agonist treatment (OAT) has been found to improve multiple health and social outcomes for individuals with OUD and has a protective effect on mortality [24–26]. Moreover, OAT has been found to reduce hospital readmissions [15, 16, 27–29]. Treatment with diacetylmorphine, or heroin-assisted treatment (HAT), is an evidence-based not-first line treatment option directed to individuals for whom standard OAT has not been sufficient. This primarily includes high-risk individuals that continue to use or inject illicit opioids or show limited improvement in physical, mental, or social functioning. Because of its target population, HAT patients often represent a particularly vulnerable group at treatment initiation [30, 31], potentially leading to elevated healthcare needs.

Collecting and using subjective and patient-reported measures has been encouraged to assess OUD treatment outcomes [32, 33]. Among others, health-related quality of life (HRQoL) measures patients’ everyday functioning, including physical and mental health limitations [33]. A 2010 systematic review found that compared to the general population and people with various medical illnesses, individuals with OUD have lower HRQoL [33]. Low scores have been found especially for mental health dimensions [33, 34]. OAT has been found to improve HRQoL over time, especially in the short-term period;, however for longer periods, the results are less clear [33, 35–38].

HRQoL measures give a subjective account of health and are often studied in relation to other (traditional) clinical outcomes. Studies in other settings have investigated the relationship between HRQoL measures and re-hospitalization and mortality, finding that lower HRQoL scores have been associated with higher healthcare utilization and increased mortality [39–46]. Nevertheless, the majority of studies were conducted over 10 years ago, and none have specifically focused on HAT patients, despite previous studies having investigated HRQoL trajectories and finding heterogeneity within the OUD patient groups [38, 47]. Although it is well established that (HR)QoL factors predict hospitalization and mortality, it is unclear which specific factors– such as social, physical, mental health - are most predictive or whether they contribute equally. By understanding these relationships, we may determine if HRQoL scores could offer prognostic information, helping to identify HAT patients at greater risk of hospital readmission and premature death. This knowledge could then inform and guide targeted interventions.

Therefore, the aims of the current study are to:

- Investigate which HRQoL factors, measured with the 36-Item Short Form Health Survey (SF-36), predict recurrent hospital-based care (HBC) (i.e., hospitalization) and mortality for patients undergoing heroin-assisted treatment in Denmark during 2010–2018.
- Identify distinct patient groups by characterizing their HRQoL trajectories over time.
- Compare average hospital contact hours and mortality rate between the identified groups and the general Danish population.

## Methods

### Setting

In Denmark, an estimated 20,000 high-risk opioid users were reported in 2023, with about 5,700 individuals receiving OAT in 2022 (nearly 6,350 when including Correctional Service data) [48, 49].

HAT has been available in Denmark since 2010 and is offered free of charge in five specialized day clinics located in different areas of Denmark. Patients self-administer heroin (injectable or tablet form) in a supervised setting up to twice daily, while oral methadone or slow-release oral morphine are prescribed for overnight maintenance or other selected periods (e.g., holidays). The treatment characteristics, clinic services, and eligibility criteria are further described in recent articles on HAT in Denmark [31, 50].

### Sample

Participants included patients from all the five existing HAT clinics in Denmark from 2010 to 2018. The observational period ranged from 22/03/2010 to 21/06/2019. Participants completed SF-36 assessments as part of the five HAT clinics' regular reporting of their patients in treatment, with the study encompassing those with at least one measurement (‘S-ALL’) and a subset with five measurements (‘SS-FIVE’). The entire sample (‘S-ALL’) comprised 541 patients (2262 assessments), and the subset (‘SS-FIVE’) included 173 patients (865 assessments). Patients without SF-36 data were implicitly excluded. A sample of the Danish general population individually matched for age, gender, and municipality was included. Registry data, linked using unique identification numbers, comprised records from various national registers (see Online Resource SI).

### Measures

The first patient assessment is filled out at the clinic together with a clinician at treatment admission, and although follow-up assessments are indicated every six months, they are typically completed annually [50]. The assessment includes the Danish SF-36 Health Survey, a widely used HRQoL measure validated for this population in a previous study [50]. When all questions have been answered, the weighted score is automatically calculated and added to the patient record. The SF-36 was selected because it routinely has been collected as part of the administrative quality monitoring at all of the five HAT clinics since 2010, allowing for a longitudinal assessment of HRQoL. The SF-36 assesses 36 items categorized into eight factors: ‘physical functioning’ (PF), ‘role limitations due to physical problems’ (RP), ‘bodily pain’ (BP), ‘general health perceptions’ (GH), ‘vitality’ (VT), ‘social functioning’ (SF), ‘role limitations due to emotional problems’ (RE), and ‘general mental health’ (MH); higher scores indicate better health in each factor [51]. To best estimate the factor scores in the representative sample, we used the most recent measure of HRQoL score measured using the SF-36 for a Danish population survey (the year 2005) [52].

Additional data included gender, age, and hospital-based contacts, which were retrieved from national registers. Hospital-based contact was defined as any registered inpatient and outpatient visit to a public or private hospital for any ICD-10 code, with the exception of mammograms. The subset psychiatric hospital contacts refers to diagnosis within the F-codes of the ICD-10 diagnostic codes (F20-F59, F340-411, and F603-F608). To account for hospitalization burden, we created a variable reflecting contact duration: outpatient visits were assigned one hour, and inpatient stays 24 hours—a necessary approximation due to the lack of precise duration data in national registers (more information in Online Resources SI).

### Analytical strategy

Predictors of recurrent HBC were analyzed using recurrent event regression [53], using the joint Cox model [54] to estimate recurrent events (i.e., HBC) and terminal events (i.e., death) separately, and employing a nonparametric bootstrap method to estimate standard errors. This considered gender, age, and time-varying SF-36 factors, employing a semiparametric estimation procedure. Additionally, we performed a separate analysis consisting only of psychiatric contacts to examine potentially different prediction results, considering the possible effect of different factors related to mental health (see Online Resource SI and S1).

We employed latent class growth analysis (LCGA) to estimate potential “latent classes” or “subgroups” in Danish HAT patients and their trajectories throughout the treatment of factors significantly predicting recurrent events identified in the previous analysis. Bayesian Information Criterion (BIC) was used to determine the number of latent classes using a ‘local minima’ analysis [55] as well as clinical criteria, i.e., adhering to the aim of finding clinically relevant and meaningful subpopulations [56]. After identifying the latent classes, we employed regression analysis using linear mixed-effects models to determine the trajectory in terms of whether the HRQoL factors previously found as predictive significantly decreased, increased, or remained stable (thus defining declining, increasing, or stable class trajectories). In addition, we also considered whether the same trajectories of classes were present in the ‘SS-FIVE’ subsample, which all completed the first five questionnaires.

We calculated summary statistics for each class to provide a descriptive statistical account of findings from the analyses mentioned above. Here, we calculated each class’s average hospitalization hours and mortality while comparing them to a demographically matched representative sample. We counted outpatient and inpatient hospital contact hours. The sum was divided by the number of hours a given patient was observed from the start of treatment enrollment to the end of the observational period, thus arriving at the average hospitalization hours for that patient. We further tracked the demographically matched sample for the same length of time as each matched patient and counted the number of hospitalization hours and terminal events. We provide descriptive plots of the average hospitalization hours and mortality. We also compared the time tracked and initial age of all four classes and representative sample (see Online Resource SI and S4 for more details on the entire analytical strategy).

## Results

Of the 541 HAT patients included in the study, the majority were male (74%, *n* = 399), and the patient ages ranged from 21 to 65, with an average age of 41.4 (SD = 8.9).

### Recurrent event regression of SF-36 factors onto hospitalization contacts

In the recurrent event regression analyses, we found that only scores on the ‘general health’ factor significantly predicted recurrent HBC (Est = -0.65, SE = 0.22, Z=-3.01, *p* =.002). As expected, the relationship was negative, such that lower ‘general health’ was related to a greater amount of future recurrent hospitalization (see Table 1). For terminal events, we observed that older patients had a higher likelihood of a terminal event (Est = 0.07, SE = 0.20, Z = 3.69, *p* <.001). Likewise, we observed that lower scores on the ‘role limitations due to emotional problems’ factor were related to a higher likelihood of a terminal event (Est = -1.18, SE = 0.60, Z= -1.96, *p* = 0.496). We found no significant predictors of recurrent psychiatric hospitalization and terminal events in the separate analyses using only mental health diagnoses (see Online Resource S1).

When distinguishing between inpatient and outpatient hospital contacts (Online Resource S5), we found that the main effect of the model is primarily driven by outpatient contacts, in which ‘general health’ has a high predictive statistical significance. In terms of inpatient contacts, these were significantly predicted by ‘vitality’, which shows a positive correlation, and the variable ‘mental health’ which had a negative correlation with the frequency of inpatient contacts.

**Table 1:** Results from the recurrent event regression with a list of parameter estimates for regressors of gender, age, and SF-36 HRQoL measures as predicting future recurrent hospital-based care (inpatient and outpatient) and terminal events, (*n* = 541)

Recurrent Event Process:						Terminal Event:				
Variable	Estimate	StdErr	z.value	*p*.value		Variable	Estimate	StdErr	z.value	*p*.value
Gender	0.260	0.21	1.20	0.240		Gender	0.405	0.36	1.13	0.258
Age	-0.001	0.11	-0.12	0.905		**Age**	**0.072**	**0.20**	**3.69**	0.000***
Physical Functioning (PF)	-0.290	0.27	-1.08	0.278		PF	-0.250	0.62	-0.40	0.685
Role Physical (RP)	0.336	0.43	0.78	0.436		RP	0.311	0.86	0.36	0.716
Bodily Pain (BP)	0.126	0.10	1.22	0.22		BP	0.300	0.22	1.33	0.181
**General Health (GH)**	**-0.654**	**0.22**	**-3.01**	0.002**		GH	-0.635	0.49	-1.29	0.197
Vitality (VT)	0.491	0.33	1.48	0.138		VT	0.543	0.44	1.24	0.215
Social Functioning (SF)	0.128	0.18	0.71	0.479		SF	-0.410	0.36	-1.14	0.256
Role Emotional (RE)	-0.113	0.38	-0.30	0.766		**RE**	**-1.185**	**0.60**	**-1.96**	0.049*
Mental Health (MH)	-0.518	0.35	-1.49	0.136		MH	0.219	0.61	0.36	0.719

### Latent class growth analysis of HRQoL factors trajectories

Because ‘general health’ was the only SF-36 factor predictive of recurrent HBC, we explored the “latent classes” that changed or remained stable in levels of ‘general health’ over time. When determining the number of classes using LCGA, we found that BIC improved with each class, which is a common finding with LCGA analyses. Here, the breakpoint analysis showed that 2.345 is the only significant breakpoint for *n* classes, meaning that more than 2 classes substantially improve the fit of the LCGA analysis (the local minimum BIC). We, therefore, explored a three-class model and a four-class model and compared them to the subsample with at least five measurements [50]. We found that the four-class model had the same trajectories of ‘general health’ for the ‘S-ALL’ sample and the ‘SS-FIVE’ subsample, whereas the three-class model did not generalize its trajectories from the subsample to the total sample; therefore, we decided on a four-class model (More information in Online Resource S2).

**Fig. 1:**
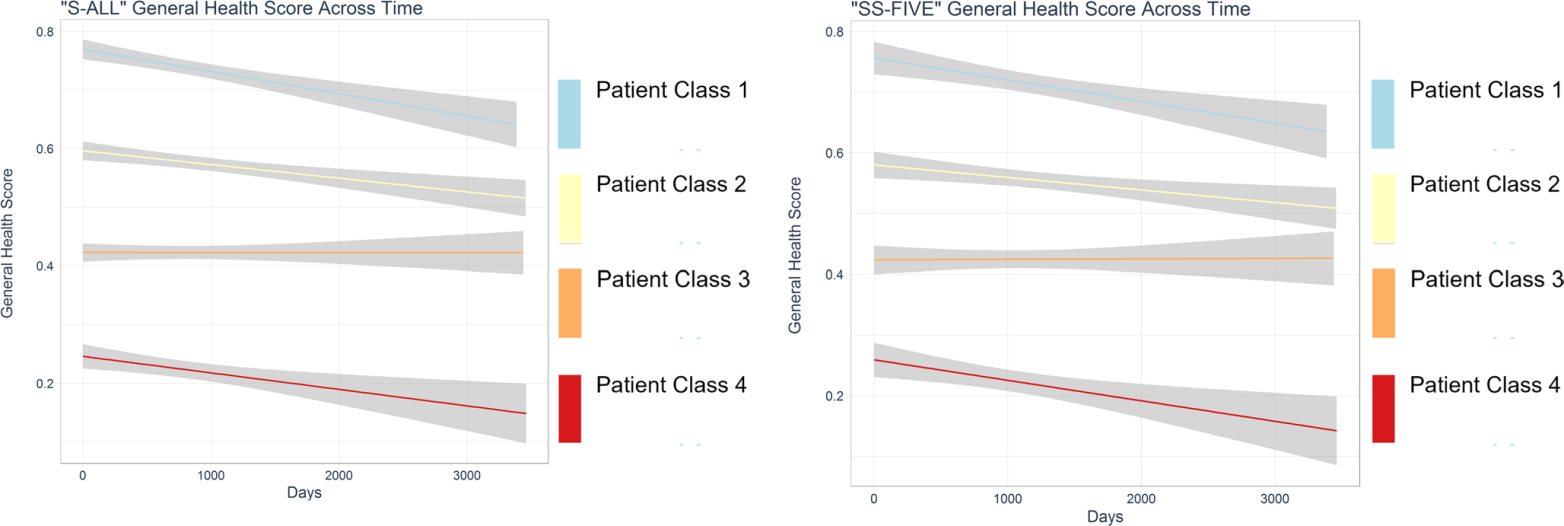
Development trajectories of four latent trajectory classes of HAT patients. Figure 1. a refers to the total sample S-ALL (*n* = 541), Fig. 1. b describes subsample SS-FIVE (*n* = 173)

The treatment trajectories and statistical significance of the four latent classes are presented in Fig. 1; Table 2 (see Online Resource S2 for more detail). For trajectories classes 1, 2, and 4, which accounted for 24%, 30%, and 13% of total patients’ membership, a significant decrease in ‘general health’ scores over treatment was noted (*p* <.001). For class trajectory 3, which accounted for the largest group (33% of the sample), the ‘general health’ score did not differ significantly over time (slope = 0.000006, *p* =.75). Taking into account the intercept estimate (first assessment) and the slope estimate (development over time/trajectory), the 4 groups were defined respectively: (1) High-Declining, (2) Above average-declining, (3) Below average–Maintaining, (4) Low-Declining.

**Table 2:** Class membership - Intercept and slope of each latent class (*n* = 541)

	Class Name	*N*	%	Male/ Female	Intercept Estimate	SE.	Slope Estimate	SE.
C.Traj 1	High–Declining	129	23.8%	93/36	0.83^***^	0.026	-0.0000827 ^***^	0.00002
C.Traj 2	Above average- Declining	162	29.9%	127/35	0.24^***^	0.024	-0.00005698^***^	0.00001
C.Traj 3	Below average -Maintaining	181	33.5%	128/53	-0.35^***^	0.022	0.0000060	0.00002
C.Traj 4	Low–Declining	69	12.8%	51/18	-0.90^***^	0.030	-0.0000940***	0.00002

C.Traj = Class trajectory; *p <.05, **p <.01, ***p <.001

### Average hospitalization hours and terminal events for latent classes and representative sample

**Fig. 2:**
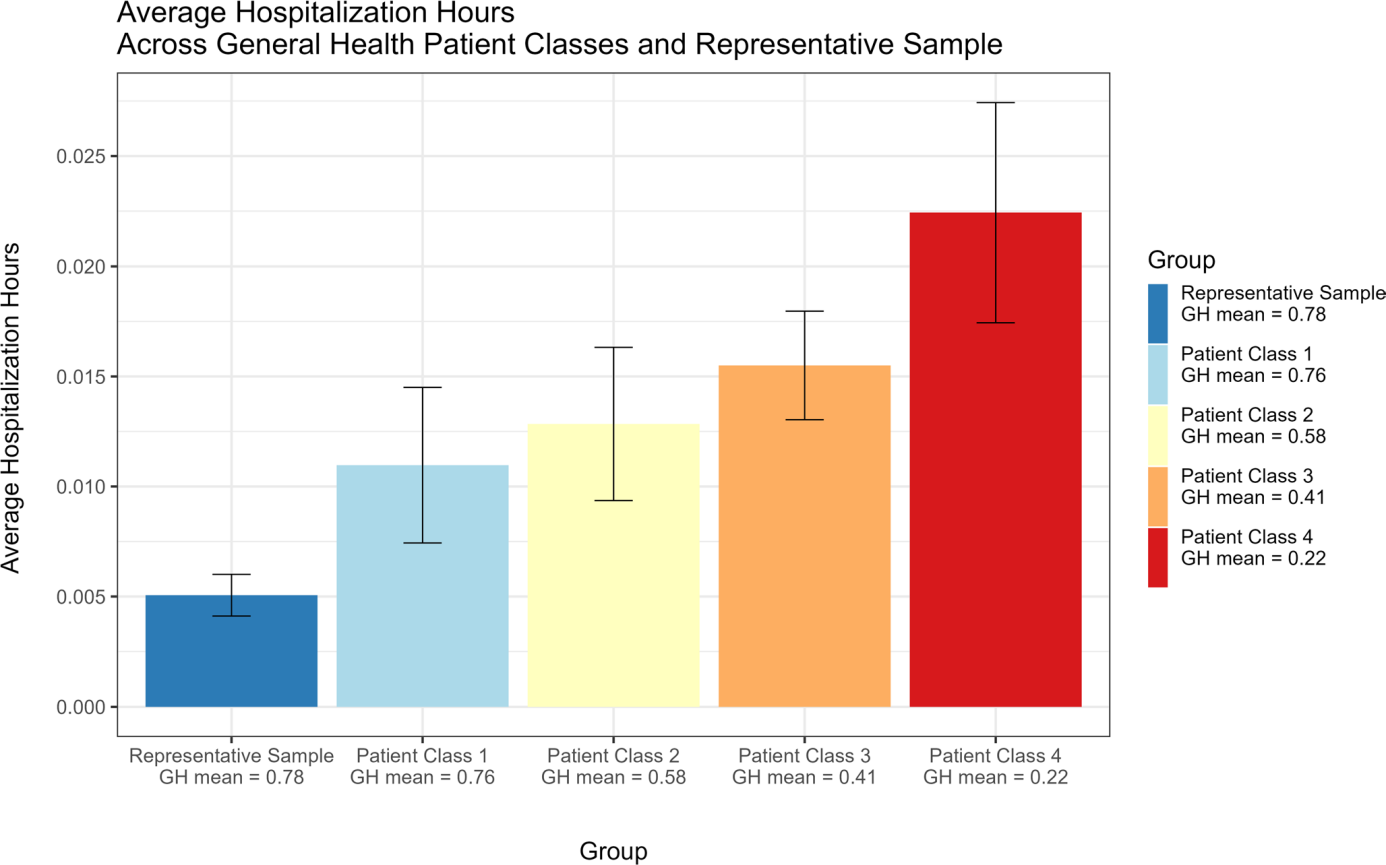
Hospitalization hours and mortality across patient classes and representative sample

Taking the representative sample (mean ‘general health’=0.78) as a reference, the estimated average hospitalization hours were higher for the four HAT patient classes, with progressively higher amounts as the classes present a lower ‘general health’ mean score (Fig. 2). With a close mean ‘general health’ score (0.74), Class 1 utilized on average double the amount (mean = 0.012) than the representative sample (0.006). While reporting the lowest ‘general health’ mean score (0.22), patient Class 4 (Low-Declining) had the highest number of hours of hospital contacts (mean = 0.023) across the observational period. To help interpret average hospitalization hours, 100 hours (roughly 4 days) with 2 hours of hospitalization equals 0.02 average hospitalization hours, whereas half an hour corresponds to 0.005, which was the average of the representative sample.

**Fig. 3:**
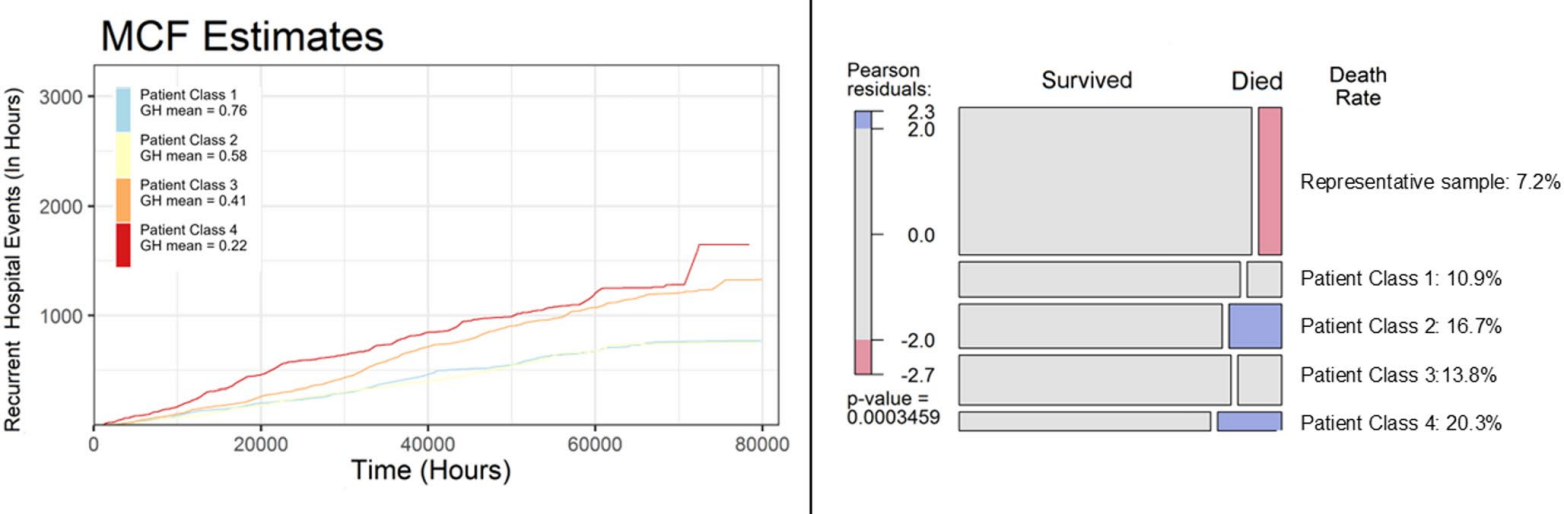
Hospitalization hours and mortality across patient classes and representative sample. **Left**: Mean Cumulative Function (MCF) estimates of recurrent hospital events (in hours) across the four different latent patient classes defined by the previous latent class growth analysis. Patient Class 1 (Class Trajectory 1: High– Declining), Patient Class 2 (Class Trajectory 2: Above average– Declining), Patient Class 3 (Class Trajectory 3: Below average– Maintaining), Patient Class 4 (Class trajectory 4: Low-Declining). **Right**: Mosaic plot of the number of individuals who survived or died over the observational period in the five different groups. Colors represent the level of the residual for that cell (Pearson residuals); blue indicates that there are more (red indicates fewer) terminal event observations than would be expected under the null model of an equal number of terminal events in each group

The number of hours individuals were tracked and the initial age of the representative sample were not significantly different across the representative sample and the four patient classes (see Online Resource S4). In contrast, there were significant differences in mortality across the groups as indicated by a Chi-square test (X^2^ (4, *N* = 1082) = 20.81, *p*-value = 0.0003), confirming that the distribution of terminal events depends on class membership. Visual inspection of the Pearson’s residuals in the Mosaic plot (Fig. 3), shows that class trajectory 2 (Above average - Declining) and class trajectory 4 (Low-Declining) had significantly higher mortality rates (16.7% and 20.3%, respectively), exceeding what would be expected if mortality were equally distributed across groups based on their size. In contrast, the representative sample had fewer individuals who died (7.2%).

Average hospitalization hours are defined as the number of hours using the hospital (hospital contacts and hospital visits) divided by the hours tracked over the observational period for the five groups. GH mean is the ‘general health’ mean score across all time points taken from the automatic scores calculated by the Danish Health Authority for patients or inferred from the latest SF-36 survey of Danes (year 2005) for the representative Danish sample.

## Discussion

The present study identified that the SF-36 HRQoL’s ‘general health’ factor was a significant predictor of reocurring hospital-based contacts among HAT patients, especially for outpatient contacts. While mortality was expectedly predicted by age, it was also predicted by SF-36 HRQoL’s ‘role limitations due to emotional problems’ factor. Latent class growth analysis identified four distinct trajectories of ‘general health’, each with unique starting points. Three classes had decreasing ‘general health’ trajectories (high, above-average, and low starting points), and one had a stable ‘general health’ trajectory (below-average starting point), which also had the highest class membership at 33%.

While direct comparison of our results with previous studies is not possible, prior research on individuals with OUD found three HRQoL trajectories groups: two stable and one with increasing QoL were identified by Nosyk et al. [38], while Krebs et al. describe three stable trajectories [47].

Descriptively, we could see that the average hospital hours varied parametrically as a function of class membership, with the HAT patients in the low GH starting point having the greatest amount of hospitalization hours and the high GH starting point class having fewer hospitalization hours, albeit still significantly greater than individuals in a representative Danish sample. Mortality also reflected the gradient in the GH trajectories, with patients belonging to Class 4 having a mortality of 20.3%. Both in terms of average hours and mortality rates, all the classes showed worse outcomes than the demographically equivalent sample.

The SF-36 ‘general health’ factor was the only factor predictive of recurrent hospitalization and may be conceptualized as a screening or “red-flag” tool to identify a likely need for enhanced healthcare. Other studies in other settings have found the summary measure'physical component summary' predictive of hospitalization, with low scores increasing the hazard and risk ratio [39–43, 45]. We did not, however, find any factor of the summary measure'mental component summary' score significant, which is different from other studies [39, 41, 43–45]. While our findings align partially with previous research, they highlight a more nuanced understanding of the predictive value that the ‘general health’ factor as a standalone measure, with implications for research and clinical practice (e.g., potential prognostic tool). While broad interventions are often advocated and may target overall HRQoL, our results suggest that they more likely to be successful if they specifically target improvements in ‘general health’. Future research, particularly through RCTs, should explore interventions explicitly designed to improve aspects connected with the SF-36 ‘general health’ concept and further validate our findings with actionable strategies. Additionally, general health should be considered a potential critical mediator of treatment effects, warranting further investigation.

Concerning mortality, age was highly predictive which aligns with high mortality among the older OUD population [57, 58]. As older OAT patients experience the effects of prolonged drug use, they face complex medical issues, and previous studies indicate elevated rates of premature deaths attributed to common physical causes, such as cancer and cardiovascular diseases [9, 59, 60]. Among the SF-36 factors, ‘role limitations due to emotional problems’ (i.e., decreased productivity and diligence) was a significant predictor of mortality, albeit borderline non-significant. Further studies should investigate the extent to which findings can be replicated before conclusions can be made about the involvement of this SF-36 factor in risk of mortality.

From our results on average hospitalization hours, we found that the entire HAT group utilized substantially more health services compared to the matched representative Danish sample. Despite having a similar GH mean score (0.78 vs. 0.76), the patients who were “most well” (Class 1) used on average hospital inpatient and outpatient care more than double the amount of the representative sample. Those who report the lowest GH (Class 4) used more than four times the representative sample. Within the HAT patients, Class 4 also differed significantly from the others with more use of hospital services, indicating an elevated need for care and enhanced support. Class 4 (GH ≤ 0.25) might intuitively be expected to improve the most, either due to regression to the mean or treatment effects. However, our data did not support this, raising clinical concern. Clinicians should pay close attention to those patients who may belong to Class 4, which points to an urgent need for health monitoring and provision of adequate treatment, likely across a variety of disorders, during their HAT participation. Interventional studies are urgently needed to test whether tailored strategies can alter this trajectory.

Mortality followed a similar pattern to hospitalization: membership in lower ‘general health’ groups was related to high mortality rate, with patients belonging to Class 4 having double the rate of Class 1 patients (20.3% vs. 10.9%). However, patients in Class 3 did not have a mortality rate higher than those in Class 2. This points to a nuanced interpretation: trajectory stability, even at lower levels of GH, may offer a protective effect, whereas a high baseline with subsequent decline may represent other underlying problematic dynamics. This finding warrants further exploration to determine whether maintaining health stability could be a feasible goal for certain patients when health improvements are unlikely. Overall, compared to the representative sample, HAT patients face an elevated risk of premature mortality, a finding consistent with a previous German HAT study [61].

For most HAT patients in the study, ‘general health’ scores deteriorated with time, indicating a worsening of their health burden even while in HAT. While this decline is concerning, it is not unexpected, particularly since patients were observed for a considerable amount of time and HAT serves individuals with long-term consequences of prolonged OUD, comorbidities, and aging. Although HAT offers potential benefits, the level of care provided may not always be sufficient to restore or maintain patients’ health stability, hindering substantial improvements. Stability may have different meanings and implications across groups and should not imply uniform care. For those with persistently poor health, the treatment goal may shift toward alleviating symptoms, preserving function, and QoL. In such cases, stability itself can be a meaningful clinical outcome, calling for tailored care strategies. However, prioritizing stability over improvement also raises important ethical and resource allocation considerations that future research and policy planning must address.

The SF-36 GH reflects individuals’ perceptions of their health in relation to others rather than of objective clinical status, making it sensitive to subjective experiences and illness insights. The absence of an ‘improving’ trajectory, and the stability in one group, raises questions about the limitations of care, expectations of recovery, and health self-perception in long-term HAT participants. Moreover, those who experience significant GH improvement may transition to less demanding treatment settings than HAT, and, therefore, are not represented in this study. As a result, the sample may disproportionally reflect patients with ongoing or worsening needs.

The elevated healthcare utilization observed among HAT patients, mainly driven by outpatient services and somatic diagnoses, should be interpreted in context. While high HBC may indicate significant health needs, the high number of healthcare contacts is not inherently a negative outcome; HAT patients may have increased HBC because of a structured system (e.g., referrals, point of contact, etc.) that enables them to improve their previously unaddressed health needs. This is in line with recent studies that reported that HAT daily attendance facilitates the provision of health services by lowering the access threshold [62, 63]. Moreover, this study underlines the potential of strengthening primary and preventative care in outpatient settings, including at HAT clinics, especially for patients with poorer mental health, in order to reduce unplanned healthcare interactions and overnight hospital stays [64]. The latter may employ staff who are ill-equipped to deal with this specific patient population, while the HAT setting has been shown to provide a supportive environment [63, 65–67].

While “mental health” was not a direct predictor of recurrent hospitalization, lower scores predicted higher recurrent inpatient contacts. Although we cannot determine if mental health predicts reocurring somatic or psychiatric inpatient hospital utilization, nearly one-third of tracked inpatient hours are for mental health diagnoses. As individuals with active or untreated severe psychiatric disorders are not eligible for HAT [31], the high number of inpatient contacts likely indicates the intensity of their need at the time. Therefore, it could be considered positive that these patients are engaging with the healthcare system for structured care.

Forecasting an expansion of the older OUD population, which exhibits heightened levels of health conditions and burden, an increase in demand for specific age-related health services is expected [68, 69]. An increasingly older HAT population with comorbidities has been reported [70]; therefore, health providers should potentially focus not only on general physical health but also age-informed health care including specific geriatric care.

### Limitations

Our study comes with limitations. There is a self-selection bias inherent to the sample, as it includes only patients who remained in treatment. Moreover, the SF-36 data collection occurs during doctor visits, which may not accurately represent the patient’s overall health or functioning. While some visits may coincide with periods of illness or poor functioning, patients often prefer maintaining their treatment regimens as usual, prompting appointments on average or good days.

Another limitation is the unknown nature of the healthcare utilization, which may either reflect better self-care or greater illness severity or reflect ageing and “normal health decline” making the study implications less clear. Also, HBC estimation was approximated due to registry limits. Moreover, the general population SF-36 data were collected in 2005, whereas the comparison data for hospitalizations and mortality cover 2010–2018. This mismatch in data periods, as well as potential changes in healthcare delivery and population health, limits the strength of direct comparisons. Lastly, this study takes advantage of data as part of quality insurance and for research purposes, not specifically collected for its aims, therefore it may be subject to missing data, changes in recording practices over time, and unmeasured confounding variables that we cannot account for.

## Conclusions

In our cohort of people receiving heroin-assisted treatment, patient-reported scoring on the SF-36 ‘general health’ factor was a reliable predictor of recurrent hospitalization. SF-36 HRQoL, and in particular ‘general health’ scoring, can be used to stratify risk for rehospitalization and premature mortality among HAT patients.

## Electronic supplementary material

Below is the link to the electronic supplementary material.

Supplementary Material 1

Supplementary Material 2

Supplementary Material 3

Supplementary Material 4

Supplementary Material 5

Supplementary Material 6

Supplementary Material 7

Supplementary Material 8
