# Supplementary material for: Health-related quality of life measures as predictors for recurrent hospitalization and mortality among patients in heroin-assisted treatment
Source: Qual Life Res. 2025 Jul 16;34(12):3365–76. doi: 10.1007/s11136-025-04019-5 (PMC12689800; doi:10.1007/s11136-025-04019-5)
Supplement: Supplementary file 7 — Supplementary Material 7 [file 11136_2025_4019_MOESM7_ESM.docx]

# Supplementary material – Health-related quality of life measures as predictors for recurrent hospitalization and mortality among patients in heroin-assisted treatment. Quality of Life Research

# Melis, F., Kvamme L.K., Tjagvad, C., Eide, D., Thylstrup, B., Clausen, T., Hesse, M. [francesca.melis@medisin.uio.no]

## Registry

Registry data was linked to the patient information using their uniquely assigned identification number. All individuals from HAT clinics that completed at least one SF-36 measurement are included in the study, and by definition its essential their presence in the HAT registry and Population Statistics Register; it was however not considered an exclusion criteria if their information did not link or appear to in all of the registries here listed.

The following registries were linked on a person-level information: the Population Statistics Register, the Heroin-Assisted Treatment Registry, the Registry for Causes of Death, the National Patient Register, and the Psychiatric Central Research Register [1]. The latter two record hospital admissions, discharge dates, and International Classification of Diseases 10th Revision (ICD-10) codes. Information about the Danish general population was retrieved from Danish Statistics, and their SF-36 score is inferred from the most recent Danish population survey.

**Sample**

S-ALL = 541 individuals and 2262 assessment, all the available “waves” of data collection are taken into consideration.

|  | n |  | n |  | n |
| --- | --- | --- | --- | --- | --- |
| Wave 1 | 541 | Wave 7 | 109 | Wave 13 | 26 |
| Wave 2 | 400 | Wave 8 | 100 | Wave 14 | 8 |
| Wave 3 | 307 | Wave 9 | 83 | Wave 15 | 7 |
| Wave 4 | 220 | Wave 10 | 64 | Wave 16 | 6 |
| Wave 5 | 173 | Wave 11 | 51 |  |  |
| Wave 6 | 133 | Wave 12 | 34 | Sum | 2262 |

SS-FIVE " uses only patients with all 5 "waves" and only those five waves (N = 173 individuals,  173*5=865 observations).

## Measures

Information about the patient's gender and age at enrollment and information about patients' hospital-based contacts was retrieved from the National Patient Register and the Psychiatric Central Research Register. Hospital-based contact was defined as any registered inpatient and outpatient visit to a public or private hospital for any ICD-10 code, with the exception of mammograms. The subset psychiatric hospital contacts refer to diagnosis within the F-codes of the ICD-10 diagnostic codes (F20-F59, F340-411, and F603-F608). In addition to the hospital contacts count, we created a variable considering the length of contact in terms of hours: an outpatient hospital contact accounted for one hour, while an inpatient hospital visit was 24 hours. While precise durations of inpatient stays or consultations (outpatient contact) could vary (e.g. 15, 30 – 60 minutes etc.) we needed an operationalizable time variable, ensuring consistent application of this definition across all participants and systematic measurement.

Information about hospitalization hours and terminal events are collected in the same way for patients and general population. Hospitalization and death data comes from Sundhedsregister (e.g., LPR, CPR, Cause of Death Register).

### Recurrent Event Regression of SF-36 factors onto hospital contacts

We employed a recurrent event regression approach to analyze predictors of recurrent hospitalization. For the recurrent event regression, we used the variables of gender and age at enrollment and the eight SF-36 factors measured in a time-varying fashion, which were updated throughout the observational period. These covariates were regressed onto the joint-frailty scale-change model estimated by the *reReg* R package [2]. The model accounts for joint frailty of the rate function of recurrent events and the hazard function of failure time using a semiparametric estimation procedure that does not require prior knowledge about the frailty distribution. The dependence between recurrent and failure events was left unspecified to accommodate informative censoring. We employed a joint Cox model of Huang and Wang [3] to estimate recurrent events and terminal events separately and a nonparametric bootstrap method using the default of 200 permutations to estimate standard errors of parameter estimates. Moreover, we performed a separate analysis using only psychiatry code contacts.

### Latent class growth analysis of HRQoL factors trajectories

To estimate the potential "latent classes" in Danish HAT patients throughout their treatment, we employed latent class growth analysis (LCGA). The main objective of LCGA is to examine inter-individual differences in intra-individual changes over time within a certain population [4-6]. We used the *lcmm* R package with 100 repetitions and 10 max iterations to estimate LCGA models using one to seven classes.

We used the Bayesian Information Criterion (BIC) to determine the number of latent classes. We considered both a low BIC value (with additional "local minima" analysis [7]) as well as clinical criteria, i.e., adhere to the aim of inferring clinically relevant and meaningful subpopulations [8]. After identifying the latent classes, we used regression analysis to determine the trajectory in terms of whether the HRQoL factors previously found as predictive significantly decreased, increased, or remained stable (thus defining declining, increasing, or stable class trajectories). In addition, we also considered whether the same trajectories of classes were present in the "SS-FIVE" subsample found in a previous sample (see the comparison between supplementary material S2 and S3) [9]. By ensuring there is correspondence between the entire sample and the subsample completing all five questionnaires, we provide evidence that our definition of classes is not biased by the degree to which certain classes fill out the questionnaire. We fitted linear mixed-effects models using restricted maximum likelihood estimation, partialing out the random effect of the individual over time (in days) while accounting for temporal autocorrelation (corAR1) using the *nlme* R package [10-12].

### Calculation of Average Hospitalization Hours for Latent Classes

After determining latent classes and their trajectories, we evaluated the average hospitalization hours for each class. In the Danish National Patient Register, patients enrolled in a treatment course typically visit a doctor every week, so for outpatient treatment courses, we filled one outpatient visit every seven days until the treatment course was over. We counted outpatient and inpatient hospital contact hours. The sum was divided by the number of hours a given patient was observed from the start of treatment enrollment to the end of the observational period, thus arriving at the average hospitalization hours for that patient. We compared the latent classes to the demographically matched Danish sample. We tracked the demographically matched sample for the same length of time as each patient and counted the number of hospitalization hours and terminal events. Supplementary output has a details in terms of hospital contacts unique events and hours, and distinction between mental health diagnoses and not.

To visualize the distribution of recurrent hospital events, we calculated Mean Cumulative Function (MCF) estimates, which represent the cumulative number of hospital hours over time for each patient class. The MCF provides a summary of the average hospital contact history across all patients in each trajectory class.

In addition, we used Mosaic plots to visually assess the distribution of terminal events across latent classes. The plots display observed and expected counts of terminal events for each class, and the shading representing the Pearson residuals indicating the the degree of deviation from expected frequencies under the null hypothesis of no association between class membership and mortality.

GUIDE TO SUPPLEMENTARY INFORMATION

| File | Analysis presented |
| --- | --- |
| S1 | "investigating the link between QoL and hospitalization"  S-ALL sample (n=541)   - Recurrent event model with hospital-based contacts and terminal event - Recurrent event model with only F diagnoses hospital-based contacts |
| S2 | Analysis in support to Figure 1 and Table 2  S-ALL sample (n=541)  Latent class analysis output and composition of the 4 selected classes  How the 4 selected classes are accounted over time (how many waves in each class)  Linear mixed-effects model fit by REML for each of the classes |
| S3 | As in S2 but using SS-FIVE (n=173) as group for analysis to confirm trajectories results. |
| S4 | Information regarding the differences among the 4 Classes and general population in terms of: SF-36 General health, Mortality rates time tracked  General health in each group  Death rates in each group (Chi Square test)  Summary mean sd time tracked (tt)  Anova of Time tracked Across the 5 groups  Anova of Initial Age Across the 5 groups  Average hospitalization hours stats |
| S5 | Analysis as S1 and S2 but with distinction between inpatient and outpatient hospital based contacts  "in patient vs out patient rereq" |
| S6 | Additional information about the inpatient and outpatient contact hours, episodes and distinction between F diagnoses and not |

1. National Centre for Register-based Research. (2023, 24.01.2023). Danish registers. from <https://ncrr.au.dk/danish-registers>

2. Chiou, S. H., Xu, G., Yan, J., & Huang, C.-Y. (2023). Regression Modeling for Recurrent Events Possibly with an Informative Terminal Event Using R Package reReg. Journal of Statistical Software, 105(5), 1 - 34.

3. Huang, C. Y., & Wang, M. C. (2004). Joint Modeling and Estimation for Recurrent Event Processes and Failure Time Data. J Am Stat Assoc, 99(468), 1153-1165.

4. Lee, S. B., & Chung, S. (2021). The influence of multidimensional deprivation on problem drinking developmental trajectory among young adults: a longitudinal study using latent class growth analysis. Substance Abuse Treatment, Prevention, and Policy, 16, 1-11.

5. Andruff, H., Carraro, N., Thompson, A., Gaudreau, P., & Louvet, B. (2009). Latent class growth modelling: a tutorial. Tutorials in quantitative methods for psychology, 5(1), 11-24.

6. Lampousi, A.-M., Möller, J., Liang, Y., Berglind, D., & Forsell, Y. (2021). Latent class growth modelling for the evaluation of intervention outcomes: example from a physical activity intervention. Journal of Behavioral Medicine, 1-8.

7. Muggeo, V. M. (2008). Segmented: an R package to fit regression models with broken-line relationships. R news, 8(1), 20-25.

8. Nylund, K. L., Asparouhov, T., & Muthén, B. O. (2007). Deciding on the Number of Classes in Latent Class Analysis and Growth Mixture Modeling: A Monte Carlo Simulation Study. Structural Equation Modeling, 14, 535 - 569.

9. Kvamme, T. L., Thylstrup, B., & Hesse, M. (2024). Quality of life assessment in Danish Heroin Assisted Treatment Patients: Validity of the SF-36 Survey. Journal of Psychosocial Rehabilitation and Mental Health, 1-11.

10. Bolker, B. M., Brooks, M. E., Clark, C. J., Geange, S. W., Poulsen, J. R., Stevens, M. H. H., & White, J.-S. S. (2009). Generalized linear mixed models: a practical guide for ecology and evolution. Trends in ecology & evolution, 24(3), 127-135.

11. Zuur, A. F., Ieno, E. N., Walker, N. J., Saveliev, A. A., & Smith, G. M. (2009). Mixed effects models and extensions in ecology with R (Vol. 574): Springer.

12. Pinheiro, J. (2011). nlme: Linear and nonlinear mixed effects models. R package version, 3, 1.
