# Supplementary material for: Health-related quality of life measures as predictors for recurrent hospitalization and mortality among patients in heroin-assisted treatment
Source: Qual Life Res. 2025 Jul 16;34(12):3365–76. doi: 10.1007/s11136-025-04019-5 (PMC12689800; doi:10.1007/s11136-025-04019-5)
Supplement: Supplementary file 8 — Supplementary Material 8 [file 11136_2025_4019_MOESM8_ESM.docx]

The RECORD statement – checklist of items, extended from the STROBE statement, that should be reported in observational studies using routinely collected health data.

|  | Item No | STROBE items | Page Number | RECORD items | Page Number |
| --- | --- | --- | --- | --- | --- |
| **Title and abstract** | 1 | (*a*) Indicate the study’s design with a commonly used term in the title or the abstract (*b*) Provide in the abstract an informative and balanced summary of what was done and what was found | 1, Abstract | RECORD 1.1: The type of data used should be specified in the title or abstract. When possible, the name of the databases used should be included.  RECORD 1.2: If applicable, the geographic region and timeframe within which the study took place should be reported in the title or abstract.  RECORD 1.3: If linkage between databases was conducted for the study, this should be clearly stated in the title or abstract. | 1, Abstract |
|  |  |  |  |  |  |
| Introduction | | | |  |  |
| Background/rationale | 2 | Explain the scientific background and rationale for the investigation being reported | 2,3 |  |  |
| Objectives | 3 | State specific objectives, including any prespecified hypotheses | 3 |  |  |
| Methods | | | |  |  |
| Study design | 4 | Present key elements of study design early in the paper | 3 |  |  |
| Setting | 5 | Describe the setting, locations, and relevant dates, including periods of recruitment, exposure, follow-up, and data collection | 3,4 + Reference to a previous article on the same sample |  |  |
| Participants | 6 | (*a*) Give the eligibility criteria, and the sources and methods of selection of participants. Describe methods of follow-up | 3 (Sample paragraph) | RECORD 6.1: The methods of study population selection (such as codes or algorithms used to identify subjects) should be listed in detail. If this is not possible, an explanation should be provided.  RECORD 6.2: Any validation studies of the codes or algorithms used to select the population should be referenced. If validation was conducted for this study and not published elsewhere, detailed methods and results should be provided.  RECORD 6.3: If the study involved linkage of databases, consider use of a flow diagram or other graphical display to demonstrate the data linkage process, including the number of individuals with linked data at each stage. | 3 (Sample paragraph),  Validation study cited [48]  Information on linkage of databases provided in Supplementary material SI |
|  |  | (*b*) For matched studies, give matching criteria and number of exposed and unexposed |  |  |  |
| Variables | 7 | Clearly define all outcomes, exposures, predictors, potential confounders, and effect modifiers. Give diagnostic criteria, if applicable | 3-4 and Supplementary | RECORD 7.1: A complete list of codes and algorithms used to classify exposures, outcomes, confounders, and effect modifiers should be provided. If these cannot be reported, an explanation should be provided. | Not provided |
| Data sources/ measurement | 8* | For each variable of interest, give sources of data and details of methods of assessment (measurement). Describe comparability of assessment methods if there is more than one group | 3-4 and Supplementary |  |  |
| Bias | 9 | Describe any efforts to address potential sources of bias | 11 |  |  |
| Study size | 10 | Explain how the study size was arrived at | 3 |  |  |
| Quantitative variables | 11 | Explain how quantitative variables were handled in the analyses. If applicable, describe which groupings were chosen and why | 3-4 |  |  |
| Statistical methods | 12 | (*a*) Describe all statistical methods, including those used to control for confounding  (b) Describe any methods used to examine subgroups and interactions  (c) Explain how missing data were addressed  (d) If applicable, explain how loss to follow-up was addressed  (e) Describe any sensitivity analyses | 3-4 and Supplementary | RECORD 12.1: Authors should describe the extent to which the  investigators had access to the database population used to create the study population.  RECORD 12.2: Authors should provide information on the data  cleaning methods used in the study.  RECORD 12.3: State whether the study included person -level, institutional -level, or other data linkage across two or more databases. The methods of linkage and methods of linkage quality evaluation should be provided | 12.3 Supplementary SI |
|  |  |  |  |  |  |
|  |  |  |  |  |  |
|  |  |  |  |  |  |
|  |  |  |  |  |  |
| Results | | |  |  |  |
| Participants | 13* | (a) Report numbers of individuals at each stage of study—eg numbers potentially eligible, examined for eligibility, confirmed eligible, included in the study, completing follow-up, and analysed | 5-8 | RECORD 13.1: Describe in detail the selection of the persons included in the study (i.e., study population selection) including filtering based in data quality, data availability and linkage.  The selection of included persons can be described in the text and/or by means of the study flow diagram |  |
|  |  | (b) Give reasons for non-participation at each stage |  |  |  |
|  |  | (c) Consider use of a flow diagram |  |  |  |
| Descriptive data | 14* | (a) Give characteristics of study participants (eg demographic, clinical, social) and information on exposures and potential confounders | Reference to a previous article on the same sample  citation [48] |  |  |
|  |  | (b) Indicate number of participants with missing data for each variable of interest |  |  |  |
|  |  | (c) Summarise follow-up time (eg, average and total amount) |  |  |  |
| Outcome data | 15* | Report numbers of outcome events or summary measures over time | 5-8 |  |  |

| Main results | 16 | (*a*) Give unadjusted estimates and, if applicable, confounder-adjusted estimates and their precision (eg, 95% confidence interval). Make clear which confounders were adjusted for and why they were included | 5-8 and Supplementary |  |  |
| --- | --- | --- | --- | --- | --- |
|  |  | (*b*) Report category boundaries when continuous variables were categorized |  |  |  |
|  |  | (*c*) If relevant, consider translating estimates of relative risk into absolute risk for a meaningful time period |  |  |  |
| Other analyses | 17 | Report other analyses done—eg analyses of subgroups and interactions, and sensitivity analyses | 5-8 and Supplementary |  |  |
| Discussion | | | |  |  |
| Key results | 18 | Summarise key results with reference to study objectives | 9 |  |  |
| Limitations | 19 | Discuss limitations of the study, taking into account sources of potential bias or imprecision. Discuss both direction and magnitude of any potential bias | 11 (Limitations) | RECORD 19.1: Discuss the implications of using data that were not created or collected to answer the specific research question(s). Include discussion of misclassification bias, unmeasured confounding, missing data, and changing eligibility over time, as they pertain to the study being reported | Page 11 (Limitations) |
|  |  |  |  |  |  |
| Interpretation | 20 | Give a cautious overall interpretation of results considering objectives, limitations, multiplicity of analyses, results from similar studies, and other relevant evidence | 9-11 |  |  |
| Generalisability | 21 | Discuss the generalisability (external validity) of the study results |  |  |  |
| Other information | | | |  |  |
| Funding | 22 | Give the source of funding and the role of the funders for the present study and, if applicable, for the original study on which the present article is based | 1 | RECORD 22.1: Authors should provide information on how to access  any supplemental information such as the study protocol, raw data, or  programming code | Supplementary information provided online  Corresponding author email for enquiries |

*Give information separately for exposed and unexposed groups.

**Note:** An Explanation and Elaboration article discusses each checklist item and gives methodological background and published examples of transparent reporting. The STROBE checklist is best used in conjunction with this article (freely available on the Web sites of PLoS Medicine at http://www.plosmedicine.org/, Annals of Internal Medicine at http://www.annals.org/, and Epidemiology at http://www.epidem.com/). Information on the STROBE Initiative is available at http://www.strobe-statement.org.
